# Stability and activity of platinum nanoparticles in the oxygen electroreduction reaction: is size or uniformity of primary importance?

**DOI:** 10.3762/bjnano.12.49

**Published:** 2021-06-29

**Authors:** Kirill O Paperzh, Anastasia A Alekseenko, Vadim A Volochaev, Ilya V Pankov, Olga A Safronenko, Vladimir E Guterman

**Affiliations:** 1 Southern Federal University, Faculty of Chemistry, 344090, Zorge st. 7, Rostov-on-Don, Russia; 2 Prometheus R&D LLC, 344091, 4g/36 Zhmaylova st., Rostov-on-Don, Russia; 3 Southern Federal University, "High-Resolution Transmission Electron Microscopy” Shared Use Center, 344090, 194/2 Stachki st., Rostov-on-Don, Russia

**Keywords:** durability, electrocatalysts, morphology control, oxygen reduction reaction, platinum nanoparticles, size distribution, spatial distribution

## Abstract

Platinum–carbon catalysts are widely used in the manufacturing of proton-exchange membrane fuel cells. Increasing Pt/C activity and stability is an urgent task and the optimization of their structure seems to be one of the possible solutions. In the present paper, Pt/C electrocatalysts containing small (2–2.6 nm) nanoparticles (NPs) of a similar size, uniformly distributed over the surface of a carbon support, were obtained by the original method of liquid-phase synthesis. A comparative study of the structural characteristics, catalytic activity in the oxygen electroreduction reaction (ORR), and durability of the synthesized catalysts, as well as their commercial analogs, was carried out. It was shown that the uniformity of the structural and morphological characteristics of Pt/C catalysts makes it possible to reduce the negative effect of the small size of NPs on their stability. As a result, the obtained catalysts were significantly superior to their commercial analogs regarding ORR activity, but not inferior to them in terms of stability.

## Introduction

Nowadays, low-temperature proton-exchange membrane fuel cells (PEMFC) are gaining a wider application. This is due to their environmental friendliness, low operating temperature, and high adaptability of specific characteristics [1–3]. The key components of PEMFC membrane–electrode assemblies (MEA) are the proton-exchange polymer membrane and porous electrode layers, in which current-forming reactions of oxygen electroreduction (ORR) and hydrogen oxidation or an organic reducing agent oxidation (e.g. methanol) occur [4–5]. The need to carry out high-rate electrode reactions requires electrocatalysts (i.e., platinum nanoparticles – NPs – or its alloys), deposited mainly onto nano/microparticles of carbon supports, which are currently the best choice [6–8]. The most important functional characteristics of the catalytic layers are their activity in the corresponding reactions and stability, which reflects the ability to maintain its activity during operation. These characteristics depend on the PEMFC operating conditions, the composition and structure of the catalytic layers, and the catalysts themselves [9].

Platinum–carbon catalysts, whose composition and structure determine their functional characteristics, are the key components of MEA catalytic layers. Of particular importance is the study of the catalyst electrochemical behavior in the ORR, since it is at the cathode that strong polarization and pronounced degradation of the catalyst take place. Such а degradation occurs as a result of both the operation at high anodic potentials and the effect of aggressive oxygen-containing intermediates, which are formed during the multistage oxygen electroreduction reaction. When comparing platinum catalysts based on the same carbon support, differences in their electrochemical behavior are determined by the difference in the composition (Pt loading in Pt/C), structure (shape and size of the platinum NPs, dispersion of their size, and features of spatial distributions), and the adhesion strength of NPs to the support [10]. The latter is likely to depend on the method/conditions of the synthesis [11].

The number of publications related to the effect of the structure of Pt/C catalysts on their activity, primarily in ORR, is rather large. Nevertheless, more and more new papers, specifying the peculiarities of such influence, are being published every year. Initially, most researchers were inclined to believe that a decrease in the size of the nanoparticles is accompanied by a significant decrease in the platinum-specific surface activity. K. Kinoshita was one of the first researchers to obtain such results [12]. As a first approximation, the specific activity of platinum in the catalyst, being referred to the unit mass of the metal *I*_mass_ (mass activity), is determined as a product of the electrochemically active surface area (ESA) and the specific activity (*I*_sp_) of platinum:

[1] $$I_{\text{mass}}=\text{ESA}\cdot I_{\text{sp}}.$$

It was found that with the increase in the ESA, the ORR mass activity of Pt/C passed through a flat maximum in the ESA values in the range of 60–90 m^2^·g^−1^(Pt), which was explained by the inverse dependence of each factor in Equation 1 on the size of platinum NPs [13]. Later, it was shown that the effect of the platinum NP size on the specific surface activity was much weaker than it seemed to be at first. For example, according to the results presented in [14], with a decrease in the average size of platinum nanoparticles (i.e., from 5–6 to 1–2 nm) the specific ORR activity of Pt/C decreased no more than two times. In this case, an increase in ESA due to a decrease in the NP size can prevail over a decrease in *I*_sp_ and, according to Equation 1, leads to an increase in the mass activity of Pt/C catalysts containing ultra-small platinum NPs. For example, in [15–16] the authors succeeded in obtaining Pt/C electrocatalysts based on ultra-small platinum NPs, which demonstrates a higher ORR mass activity in comparison with widespread commercial Pt/C analogs. In fact, different sites of NP surfaces have different specific activities in the ORR [17]. As a result, the control of the NP shape, leading to an increase in the proportion of more active areas, can lead to a significant increase in the Pt/C activity [18]. Moreover, according to the calculations in [17], NPs of each size can have its own optimal shape, which provides the highest ORR mass activity. Nevertheless, it is important to take into account that real Pt/C catalysts contain nanoparticles of different sizes. To have the control over the size of the particles can cause the change in their shape, which in turn can result in the formation of a maximum on the curves of activity dependence on the average NP size [19].

The effect of Pt/C catalysts structure on durability has been less studied than the effect on the ORR activity. To assess the stability of the catalysts, much longer tests to evaluate their activity are required. Moreover, the correlation between the results of evaluating catalyst durability obtained during accelerated stress tests and the use of fuel cells in real practice is far from being perfect. Therefore, along with the study of how certain factors influence on the catalyst stability, the search for optimal methods and conditions for stress tests in an electrochemical cell is still progress. The degradation of electrocatalysts is a highly complex process and it can proceed in accordance with different mechanisms [9]. It has been established that the regression of Pt/C functional characteristics can be associated with different reasons, including the dissolution of small platinum NPs (less than 3 nm in size) [14,20], reprecipitation of platinum from small NPs into larger ones [14,21–22], agglomeration of NPs in the process of their surface diffusion [9,22], and NP shape change [4,23]. This can happen due to the oxidation of the carbon carrier, which causes the detachment of platinum NPs and loss of contact with the support [24–26], as well as the isolation of particles by adsorbed carbon monoxide released due to carbon oxidation [27–30]. Each of the mechanisms previously described can play a greater or smaller role, depending on the conditions of the catalysts tested and the features of their composition and structure [31].

When the same support is used, the stability of Pt/C catalysts during their operation is affected primarily by the mass fraction of the metal and the size of the nanoparticles [32–33]. The catalyst stability, as a rule, increases with increasing NP size [14,22,32–36]. The probability of agglomeration and coalescence of NPs during a stress test also decreases with an increase in the average distance between platinum NPs in catalysts, which results in an increase of their stability [14]. Apparently, a relatively high stability of small-sized systems is possible when the platinum NPs are similar in size and their distribution is uniform over the surface of the carbon support [35,37–40]. For example, as it is shown in [40], the growth of the nanoparticles during the catalyst operation accelerates when large and small NPs are localized in the same regions of the support surface, and it slows down if the catalytic layer is formed from an ordinary mixture of two catalysts with small and large nanoparticles, respectively.

The need to reduce the content of expensive platinum in the catalysts forces the researchers to follow the path of reducing the size of NPs. However, the opposite effect of the NP size on the specific activity, stability, and ESA of the catalysts forces us to seek a compromise or, in other words, to seek structures with an optimal combination of catalyst mass activity and stability. In this regard, of particular interest are the methods for the synthesis of catalysts, which make it possible to obtain materials that combine small size of the nanoparticles, their narrow dimensional and uniform spatial distribution over the surface, and pores of support.

This study was based on the hypothesis that Pt/C catalysts containing small nanoparticles, which are similar in size and uniformly distributed over the surface of a carbon support, can be both more active and more stable than catalysts based on larger particles, but with less uniformity of dimensional and spatial distribution.

Thus, the aim of this study was to obtain Pt/C catalysts containing small NPs narrow in size and with uniform spatial distributions and to compare the ORR activity and stability of the obtained catalysts and conventional analogs containing NPs of a larger size, but with less uniform structural characteristics. Taking into account the above requirement, we chose commercial catalysts that are widely used both in research and in the manufacture of fuel cells.

## Experimental

### Materials

The synthesis of Pt/C catalysts was carried out in the liquid phase according to the procedure described in detail in [16]. Formaldehyde was used as a reducing agent and the synthesis was carried out in an atmosphere of carbon monoxide. A weighed portion of a Vulcan XC-72 carbon support (Cabot Corporation) weighing from 0.150 g (when obtaining the material G40) to 0.055 g (when obtaining the material G20) was introduced into 18 mL of ethylene glycol, homogenized by ultrasound for 10 min, and then stirred on a magnetic stirrer for 15 min. Then, without stopping stirring, a water solution of H_2_PtCl_6_ (TU 2612-034-00205067-2003, Pt mass fraction of 37.6%, Aurat, Russia) was introduced into the suspension and the pH was adjusted to 10 by adding a 0.5 M KOH solution (JSC Vekton, Russia). Then, 1 mL of formaldehyde (37%, JSC Vekton, Russia) was added and the suspension was purged with carbon monoxide for 15 min. Then, without stopping the CO blowing, the reaction mixture temperature was increased to 90 °C and the mixture was kept under constant stirring for 2 h. The composition of the obtained catalysts is given in Table 1. The numbers in the names of the samples correspond to the calculated platinum mass fraction in Pt/C.

**Table 1:** Parameters characterizing the composition and structure of Pt/C catalysts.

sample	Pt loading in Pt/C, ω(Pt), wt %		average crystallite diameter, *D*_Av_, nm (XRD)	average NP size, D_NP_, nm (TEM)	specific number of NPs, N, 10^15^ m^−2^	average distance between NPs, λ, nm

	theoretical	actual				

G20	20	20.4 ± 0.6	1.2 ± 0.1	2.0 ± 0.1	9.6	8.0
G25	25	24.7 ± 0.7	1.2 ± 0.1	2.2 ± 0.1	9.9	7.7
G30	30	30.9 ± 0.9	1.3 ± 0.1	2.5 ± 0.1	10.1	7.4
G35	35	34.0 ± 1.0	1.3 ± 0.1	2.6 ± 0.1	10.4	7.2
G40	40	39.0 ± 1.2	1.3 ± 0.1	2.6 ± 0.1	13.0	6.2
JM20	—	20.0 ± 0.6	2.5 ± 0.1	2.7 ± 0.1	4.5	12.2
JM40	—	40.0 ± 1.2	3.7 ± 0.1	3.7 ± 0.2	5.5	9.9

Commercial Pt/C catalysts JM20 and JM40 (HiSPEC3000, 20 wt % Pt; HiSPEC4000, 40 wt % Pt, Johnson Matthey) were used as reference samples.

### Methods to verify the composition and structure of Pt/C materials

The Pt loading in the studied samples was determined by gravimetry. Ceramic crucibles were calcined to get a constant weight at 800–850 °C. Next, they were weighed after complete cooling. Then, approx. 0.02 g of the Pt/C material was placed in crucibles and kept in a muffle furnace at 800–850 °C for 40 min. Crucibles with a non-combustible residue (Pt) were weighed after complete cooling. The difference in weight was used to determine the metal content in the sample.

To study the structural characteristics of the obtained Pt/C materials, we used the powder diffraction method. An ARL X’TRA powder diffractometer with a Bragg–Brentano geometry (θ-θ), Cu Kα radiation (λ = 0.154056 nm) at room temperature, was used to record X-ray diffraction patterns. The X-ray diffraction patterns of the samples under study were recorded in the angle range of 5 ≤ 2θ ≤ 90 degrees by the step-by-step scanning method with a detector movement step of 0.02 degrees. The X-ray diffraction patterns were processed by using the SciDavis software to properly extract the parameters of the peaks and this was of particular significance when they overlapped in the case of small-sized particles. The average platinum crystallite size *D*_Av_ was calculated using the Scherrer equation, as described in more detail in Supporting Information File 1.

The size of the platinum nanoparticles, the features related to their size, and the spatial distributions were also studied by transmission electron microscopy (TEM). The TEM images were obtained using a JEOL JEM F200 microscope (voltage 200 kV, current 12–15 μA, CFEG). To prepare a sample for measurements, 0.5 mg of the catalyst was placed into 1 mL of isopropanol and dispersed by ultrasound for 10 min. A drop of the resulting suspension was applied to a standard copper mesh with a diameter of 3.05 mm, covered with a 5–6 nm thick layer of amorphous carbon. Next, the sample was dried in air at room temperature for 60 min. The histograms of platinum nanoparticle size distribution in the catalysts were plotted to determine the size of at least 400 randomly selected particles in the TEM images in different regions of the sample.

### Electrochemical investigation

Electrochemical measurements were performed in a three-electrode cell on a VersaSTAT3 potentiostat using a rotating disk electrode (RDE) (Pine Research Instruments, USA). A saturated silver chloride electrode was used as a reference electrode. The potentials were given with regard to a reversible hydrogen electrode (RHE).

A thin, porous catalyst layer was applied to the electrode using the so-called "catalytic ink". To obtain a suspension of Pt/C catalysts (i.e., "catalytic ink"), 900 μL of isopropyl alcohol and 100 μL of a 0.5% aqueous emulsion of Nafion® polymer were added to 6 mg of each sample. Then, the suspension was dispersed with ultrasound for 15 min. Under continuous stirring, an aliquot of “ink” of 6 μL in volume was taken with a microdispenser and applied to the end of a polished and degreased glassy carbon disk with an area of 0.196 cm^2^, the exact weight of the drop was recorded. The electrode was dried in air for 20 min while rotating at 700 rpm.

Prior to measuring the ESA of the catalyst, the electrolyte was saturated with argon for 40 min. Then, 100 cycles were carried out in the potential range from 0.04 to 1.2 V relative to RHE. The potential sweep speed was 200 mV·s^−1^.

The electrochemically active surface area was determined from the cyclic voltammogram (CV) by calculating the amount of electricity consumed for desorption (*Q*_d_) and adsorption (*Q*_ad_) of atomic hydrogen, as described in more detail in Supporting Information File 1. The CV recording rate was 20 mV·s^−1^ and the potential range was 0.04–1.2 V relative to RHE.

To determine the ORR activity of the catalysts, the electrolyte was saturated with oxygen for 1 h, after which a series of voltammograms was measured in the range of 0.12–1.19 V with a linear potential sweep at a rate of 20 mV·s^−1^ at an electrode rotation speed values of 400, 900, 1600, and 2500 rpm. The values of mass (*I*_mass_) and specific activity (*I*_sp_) were calculated for a potential of 0.90 V (RHE).

The method of multiple voltammetric cycling in the potential range of 0.6–1.0 V (RHE) with a sweep rate of 100 mV·s^−1^ was chosen as a method for assessing the degree of degradation of the electrocatalysts. The cycling was carried out in a 0.1 M HClO_4_ solution saturated with argon at 25 °C for 5000 cycles. After every 500 cycles, two CVs were recorded at a potential sweep rate of 20 mV·s^−1^ in the potential range of 0.04–1.2 V, with respect to RHE, to calculate the ESA, as described previously. A detailed calculation of the catalyst stability is given in Supporting Information File 1.

## Results and Discussion

**Figure 1:**
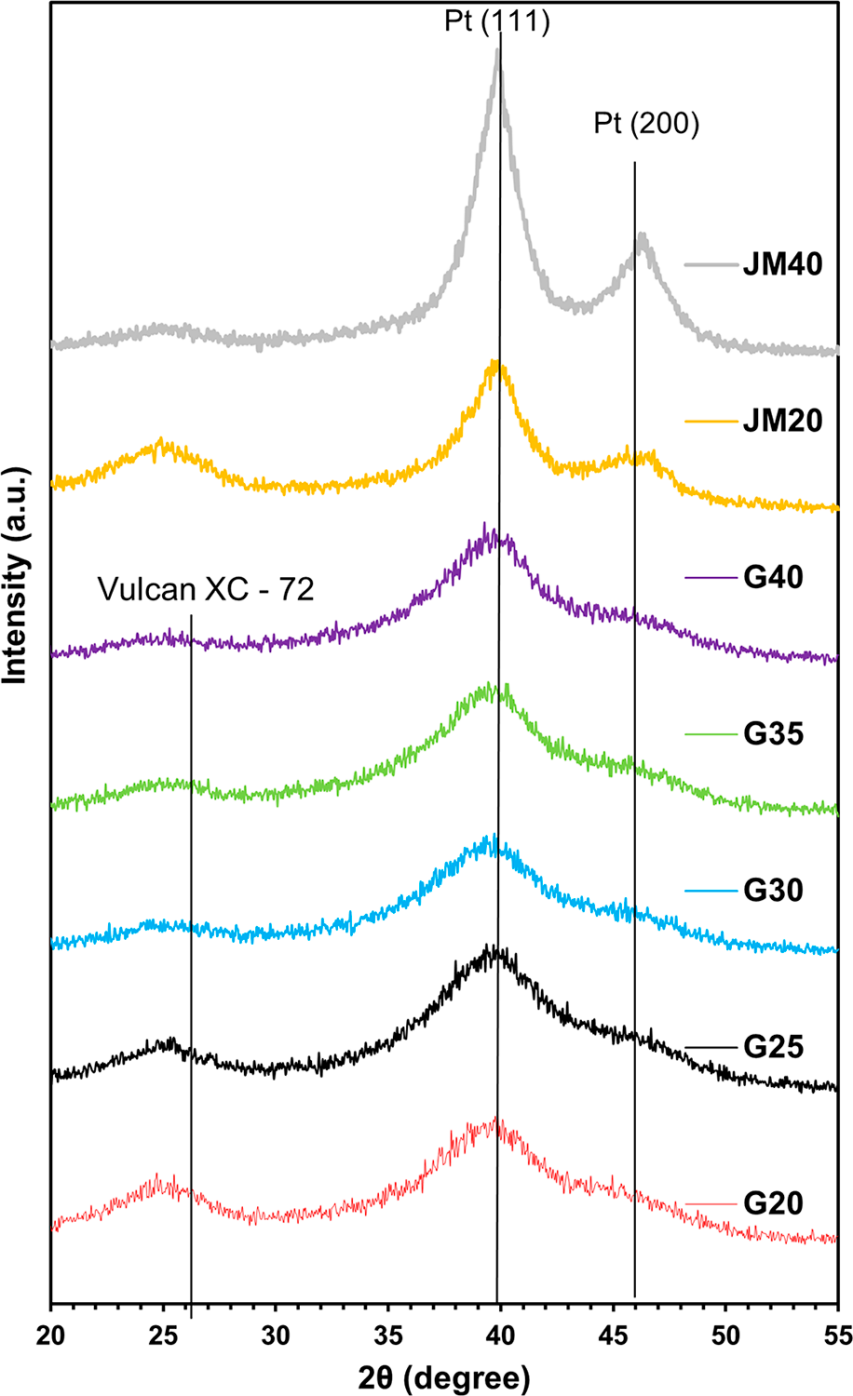
X-ray diffraction patterns of Pt/C materials.

The X-ray diffraction patterns of the catalysts that we have synthesized and the commercial analogs with different platinum contents have a similar shape, typical for Pt/C materials (Figure 1). The presence of nanosized platinum crystallites causes a broadening of the characteristic maxima of platinum. The broadening increases with a decrease in the *D*_Av_. The results of calculating the *D*_Av_ values according to the Scherrer equation are shown in Table 1.

**Figure 2:**
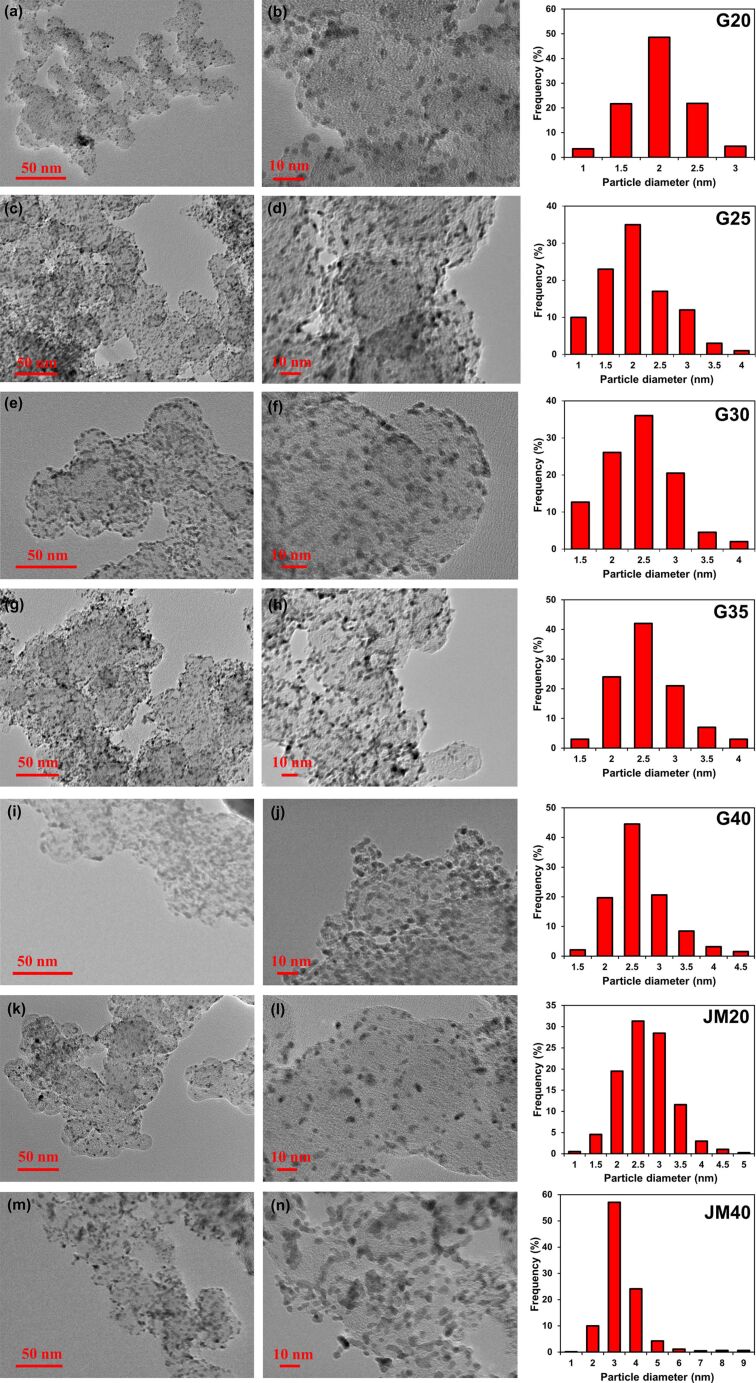
Micrographs of Pt/C samples of the catalysts G20 (a,b), G25 (c,d), G30 (e,f), G35 (g,h), G40 (I,j), JM20 (k,l), and JM40 (m,n), and histograms of nanoparticle size distribution in the corresponding materials.

The mass fraction of platinum in the obtained materials ranged from 20.4 (G20) to 39 wt % (G40). The average crystallite size in the catalysts ranged from 1.2 (samples G20 and G25) to 1.3 nm (G30, G35, and G40) (Table 2). A selective electron microscopy study of the synthesized materials (Figure 2) showed that with an increase in the Pt loading in the catalysts, the average nanoparticle size also increased from 2 (G20) to 2.6 nm (G30–G40).

**Table 2:** Parameters characterizing the electrochemical behavior of Pt/C catalysts.

sample	ESA, m^2^·g^−1^(Pt)	*E*_1/2_, V (at 1600 rpm)	number of ē	*I*_mass_, A·g^−1^(Pt)	*I*_sp_, A·m^−2^(Pt)

G20	120 ± 12	0.92	3.8	250	2.1
G25	116 ± 12	0.93	4.1	220	1.9
G30	98 ± 10	0.92	4.1	208	2.1
G35	93 ± 9	0.92	3.9	194	2.1
G40	88 ± 9	0.94	4.0	186	2.1
JM20	84 ± 8	0.91	3.9	182	2.2
JM40	65 ± 7	0.92	4.0	122	1.9

**Figure 3:**
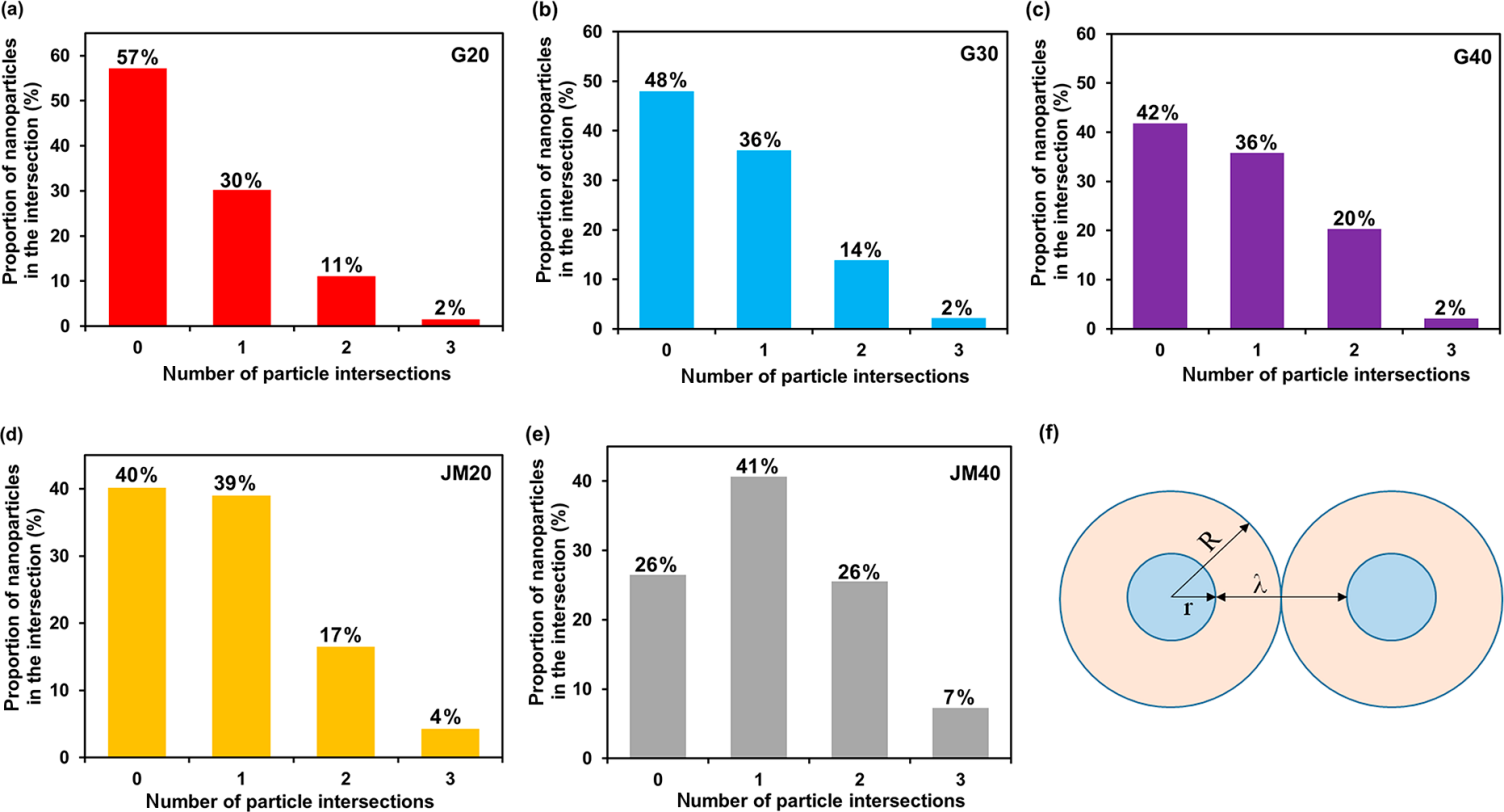
Histograms of nanoparticle distribution by the number of intersections with "neighbors" (a–e) and a schematic representation of the location of spherical nanoparticles within the geometric model (f).

The difference in the sizes of nanoparticles (crystallites) determined from the results of X-ray diffractometry and TEM is typical for nanostructured Pt/C materials. It is due to several factors: some nanoparticles can consist of several crystallites, so they have a larger size [19]; differences in the principles of calculation, which serve as the basis for the corresponding research methods [41]; a possible contribution of NP structural defects to the broadening of the X-ray diffraction pattern maxima [42], and problems related to the recognition of ultra-small particles in TEM micrographs. The composition of the commercial catalysts JM20 and JM40 is similar to that of G20 and G40: the average crystallite size is 2.5 and 3.7 nm, and the average NP size is 2.7 and 3.7 nm, respectively. The comparison of the NP size distribution histograms indicates that in the synthesized Pt/C catalysts the smaller size of NPs is combined with their size distribution, which is narrower than that in the commercial reference samples (Figure 2). For example, in the sample G20, 92% of NPs have a size of 2.0 ± 0.5 nm, while in the commercial analog JM20 they have the size of 2.75 ± 0.75 nm (Figure 3). A similar difference is observed when we compared G40 and the JM40 analog (Figure 2).

We estimated the uniformity of the spatial NP distribution over the support surface. To this end, for each Pt/M material in the TEM micrographs, we calculated the fraction of NPs that were not in contact (non-overlapping) with other NPs or in contact with one, two, or three other NPs. In this case, with the assumption that the smaller the number of intersections of NP images in the micrographs, the more uniformly distributed over the carrier surface the NPs. The disadvantages of such a simplified system used for analyzing the uniformity of the distribution are: i) the need to analyze the structure of a three-dimensional object from its two-dimensional image, and ii) the consideration of intersections, in the case when NPs are located on the lower and upper parts of the carbon particle but only their images overlap. Nevertheless, even such a comparison proves to be useful, especially in the case of comparing Pt/C materials with a similar specific number of nanoparticles per unit of surface. The histograms of the NP distribution by the number of intersections for different samples are shown in Figure 3a–e.

In the samples with the measured NP size, the average distance between the nanoparticles was calculated using a simplified geometric model. It was assumed that spherical particles of the same size are uniformly distributed on the flat surface of the carrier. Within the framework of the model, one can describe the geometry of a NP distribution by surrounding them with a region of a certain radius *R*, its value depending on the radius (*r*) of the NPs and the distance between them (λ) (Figure 3f).

To determine the values of the parameters *R* and λ, the number *N* of nanoparticles per 1 m^2^ of the carbon support was calculated:

[2] $$N_{\text{NP}}=\frac{m(\text{Pt})}{m(\text{NP})\cdot S(\text{C})},$$

where *m*(Pt) is the mass of platinum applied to 1 g of carbon support, *m*(NP) is the mass of one spherical platinum particle (g), *S*(C) is the surface area of the carbon support (m^2^·g^−1^) equals to 250 m^2^·g^−1^.

The mass of a spherical particle is determined by its size:

[3] $$m(\text{NP})=\frac{4}{3}\pi r^{3}\rho ,$$

where ρ is the density of platinum (21.45 × 10^6^ g·m^−3^). Equation 4 was used to calculate *R*:

[4] $$R=\frac{0.5}{\sqrt{N(\text{NPs})}}.$$

The average distance between nanoparticles (λ) was calculated by Equation 5:

[5] λ=2(R−r).

The results of the model calculation (Equations 2–5) of the NP specific number and the average distance between NPs for the studied catalysts are shown in Table 1.

The characteristic analysis of the platinum NP spatial distribution was carried out in accordance with the results obtained by processing the TEM micrographs (Figure 2a–e). It indicates a greater uniformity of the spatial distribution of platinum NPs in the G series catalysts as compared to the JM20 and JM40 samples. For example, in the catalysts G20 and G40, 57% and 42% of NPs, respectively, are not adjacent to their “neighbors”. At the same time, in the commercial samples of similar composition, JM20 and JM40, the proportion of NPs is only 40% and 26%, respectively. The share of NPs that has one or more intersections with "neighbors" increases in the series: G20 (43%) < G30 (52%) < G40 (58%) ≤ JM20 (60%) < JM40 (74%). It is important that, due to the large average size and average mass of NPs in commercial samples, the specific number of such particles is somewhat smaller, and the average distance between NPs, calculated within the framework of a simplified model, is slightly larger than that for NPs in the catalysts G20, G30, and G40 (Table 1). This means that there are no geometric reasons that could cause a lower uniformity of the NP spatial distribution on the surface of a carbon support in commercial Pt/C catalysts.

Thus, the original procedure used for the synthesis of Pt/C catalysts made it possible to successfully solve the first of the tasks posed in this study: to obtain Pt/C materials characterized by a smaller size of nanoparticles, narrower dimensional and more uniform spatial distribution over the surface of the Vulcan XC-72 carbon support, as compared with the commercial electrocatalysts JM20 and JM40.

**Figure 4:**
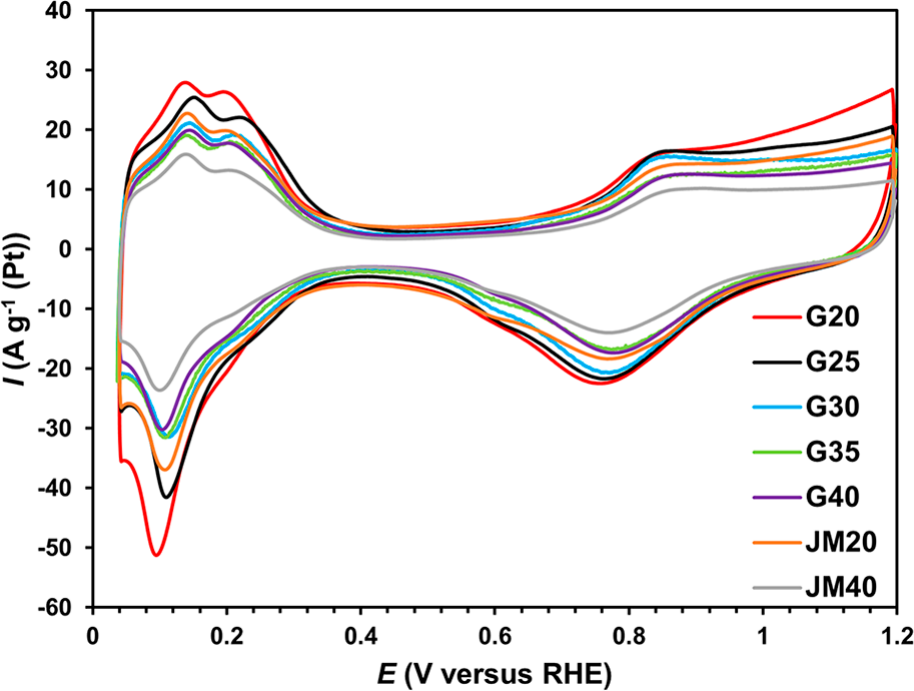
Cyclic voltammograms of Pt/C samples. Sweep rate of 20 mV·s^−1^, 2nd cycle. Electrolyte used: 0.1 M HClO_4_ solution saturated with Ar at atmospheric pressure.

The cyclic voltammograms of Pt/C catalysts have a characteristic form (Figure 4). As the content of platinum in the catalysts increases, the specific current values decrease in all sections of the CVs (Figure 4).

The calculation based on the amount of energy consumed for the electrochemical adsorption and desorption of an atomic hydrogen monolayer showed that an increase in the Pt loading in the obtained samples (from 20.4 to 39 wt %) leads to a decrease in the ESA (from 120 to 88 m^2^·g^−1^(Pt)) (Table 2), which is due to both an increase in the average size and in NP coalescence (Table 1, Figure 3).

When comparing the synthesized and commercial catalysts of the same composition, one can see that the ESA of JM catalysts is lower than that of G catalysts (Table 2), which is due to the difference in their structural characteristics described above (Table 1). The commercial catalyst JM40 has the minimal ESA value (Table 2).

**Figure 5:**
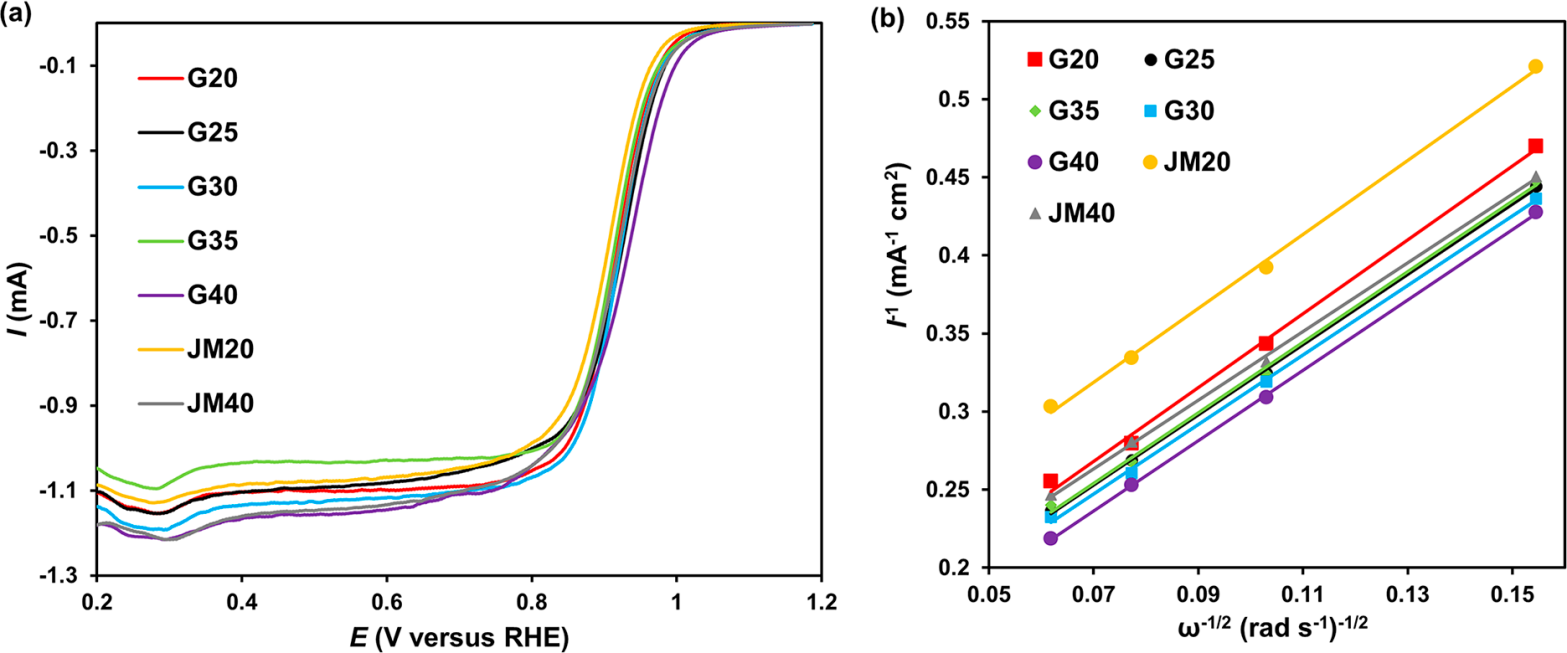
(a) Linear voltammograms of the ORR. The rotation speed of the disk is 1600 rpm^−1^, (b) *j*^−1^ - ω^−0.5^ dependence at a potential of 0.90 V. The rate of the potential sweep is 20 mV·s^−1^. Electrolyte used: 0.1 M HClO_4_ solution saturated with O_2_ at atmospheric pressure.

The study of the ORR kinetics, carried out by linear voltammetry using RDE (Figure 5), showed that the ORR proceeds according to the four-electron mechanism on all catalysts (Table 2). The values of the half-wave potential of oxygen electroreduction for the studied catalysts vary in the range from 0.91 (JM20) to 0.94 V (G40) (Table 2). Note that the values of the specific kinetic current of all catalysts are very close around 2.05 ± 0.15 A·m^−2^(Pt) (Table 2). This suggests that a change in the size of the nanoparticles in the range from 2.0 to 3.1 nm does not affect the specific activity of platinum in the ORR. The key factor that determines the mass activity of the catalysts under these conditions is the ESA. As the ESA increases in the series G40 < G35 < G30 < G25 < G20, the mass activity of the synthesized catalysts also increases (Table 2). Note that the mass activity of the sample G20 is almost 1.5 times higher than that of JM20, a commercial analog with a similar platinum content (Table 2).

**Figure 6:**
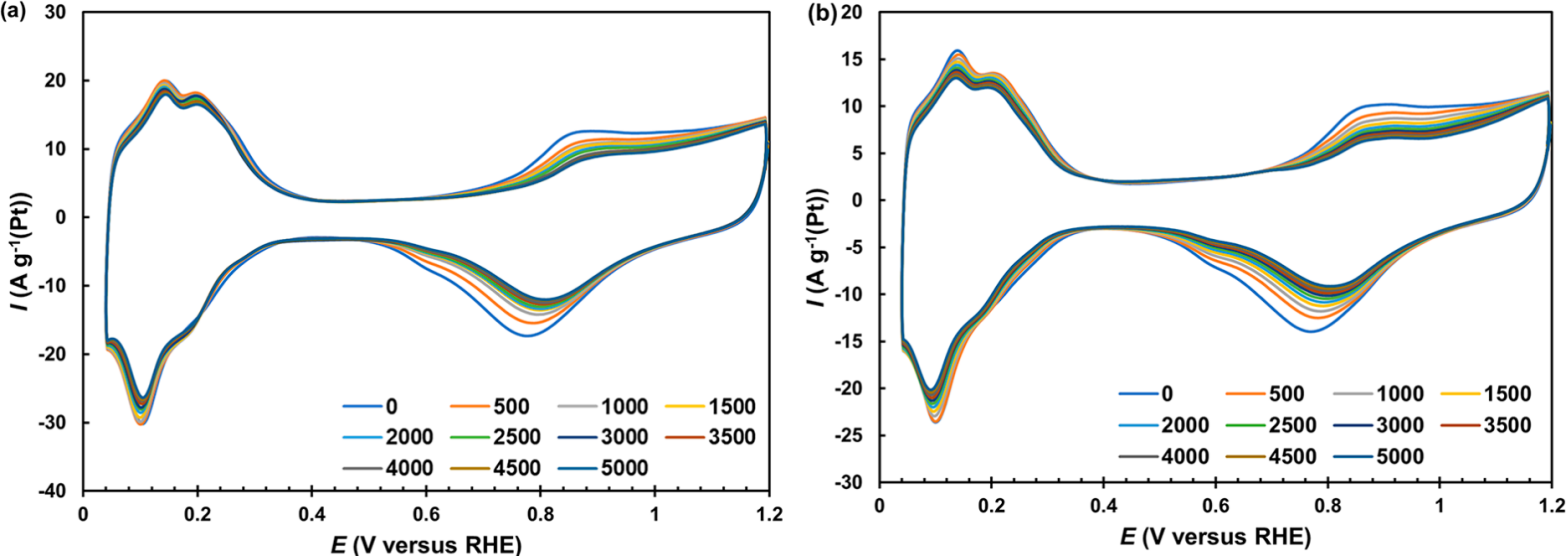
Cyclic voltammograms of (a) G40 and (b) JM40 catalysts measured at a potential sweep rate of 20 mV·s^−1^ for every 500 stress test cycles. Electrolyte is 0.1 M HClO_4_, Ar atmosphere.

During the stress test of the catalysts, a regular decrease in the current values occurs which is more pronounced in the oxygen region of the CV (Figure 6). Interestingly, the value of the relative stability of the G series catalysts was found to be the same, independent of either the mass fraction of platinum in the catalysts or the average NP size (Table 3). Despite the larger size of the platinum nanoparticles in the commercial catalysts, their relative stability turned out to be somewhat lower than that in the samples we synthesized. Apparently, the higher uniformity of the catalyst structure compensates for the negative effect of the smaller NP size on the process of their degradation. According to [14,33], the decrease in ESA and activity of the catalysts during the cycling of the potential in the range of 0.6–1.0 V is largely due to the processes of platinum reprecipitation and coalescence of NPs.

**Table 3:** Parameters characterizing the behavior of electrocatalysts upon the completion of the stress test.

sample	ESA_5000_, m^2^·g^−1^	ESA_5000_/ESA_0_, %	number ē	electric current at *E* = 0.90 V		*E*_1/2_, V	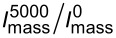 , %

				*I*_mass_, A·g^−1^(Pt)	*I*_sp_, A·m^−2^(Pt)		

G20	103	86	3.9	118	1.2	0.89	47
G25	100	86	3.8	132	1.3	0.91	60
G30	84	86	3.7	155	1.8	0.92	75
G35	80	86	3.5	145	1.8	0.92	75
G40	76	86	4.2	130	1.7	0.93	70
JM20	69	82	4.3	60	0.9	0.88	33
JM40	55	82	3.6	83	1.5	0.90	69

The proximity of the sizes of the NPs and the uniformity of their distribution should slow down the course of these negative phenomena. As we have already noted, the positive role of proximity of the sizes of NPs located in adjacent regions of the catalyst, with regard to its stability, was demonstrated in [40].

**Figure 7:**
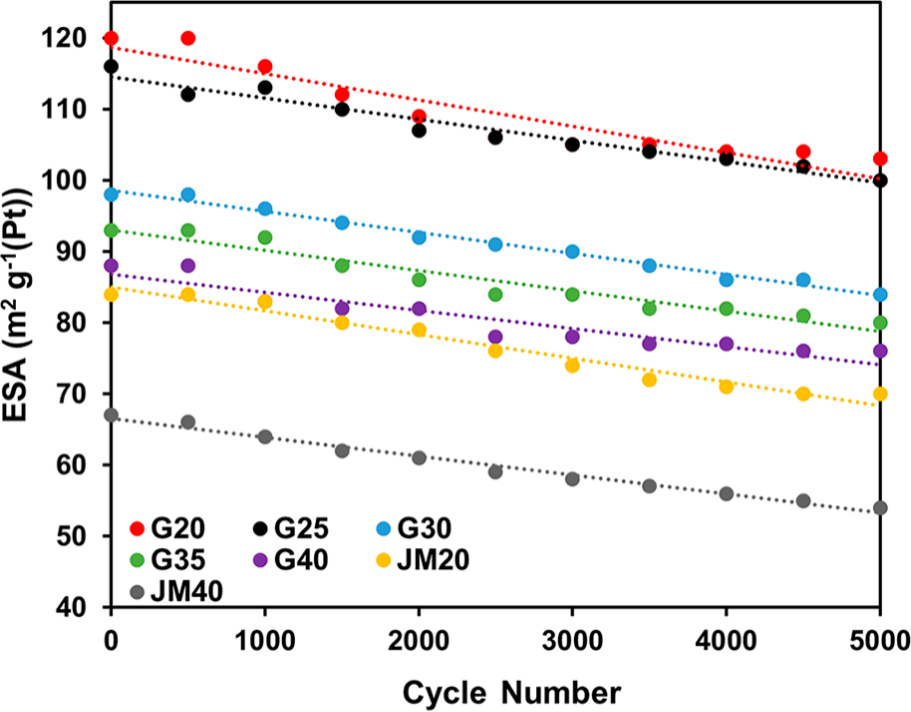
Dependence of the ESA of the catalysts on the number of stress test cycles.

Upon completion of the stress test, a decrease is observed in the specific activity of the catalysts in ORR, which is more pronounced for samples with a low platinum loading (i.e., JM20 and G20). This follows from the comparison of voltammograms (Figure 7), the values of the specific kinetic currents of ORR on the studied catalysts before (Table 2), and after (Table 3) the completion of the stress test.

The relationship between the ORR mass activity of the catalysts with specific activity and ESA can be expressed by Equation 6 [36]:

[6]
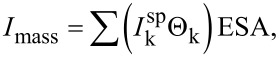


where 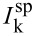
is the specific activity of the NP surface areas (faces, edges) of the k-type, Θ_k_ is the fraction of the nanoparticle surface belonging to the k-type areas. Taking into account platinum nanoparticles with different numbers of catalytically active sites on their surface (different types of faces, edges, vertices, steps) it can be assumed that the smaller the amount of platinum contained in the catalytic layer, the faster the decrease of the proportion of the most active sites during the stress test of the catalysts. Since the platinum NPs in the samples G20–G40 have a similar size, and the number of NPs in the catalytic layer increases with an increase in the platinum loading in the catalyst during the test, a much larger amount of oxygen is reduced in the catalyst G20 at each NP than that in the catalyst G40.

As a result, the reorganization of the NP structure accompanied by a decrease in the proportion of the most catalytically active faces is more pronounced in the G20 catalyst than in the G40 catalyst. At the same time, the total surface area of the NPs does not change very much because of the structural reorganization (Table 2 and Table 3), which one can see when the CVs, in their “hydrogen” region, before and after the stress test are compared (Figure 7). This determines the proximity of the relative stability values, calculated from the ESA ratio. Another reason for the platinum loading effect (the number of NPs in the catalytic layer) on the change in the catalyst activity may be the poisoning of active centers by the impurities contained in the solution and in the carbon support. Prior to the measurements, an identical amount (approx. 0.036 mg) of each catalyst was applied to the end of the disk electrode (see Experimental section). Taking into account the Pt loading in the catalysts and the corresponding ESA values (Table 1 and Table 2), it is easy to calculate that the ESA in the initially formed catalytic layers was approximately 8.8 for G20 and 12.4 cm^2^ for G40. With an identical quantity of impurities contained in the solution, the poisoning of the active centers should be more pronounced in catalyst G20 since a more significant decrease in the ORR specific activity is observed in that sample.

Upon completion of the stress test, the ORR mass activity of the catalysts increases in the following order: JM20 < JM40 < G20 < G40 ≤ G25 < G35 < G30 (Table 3). The change in the form of the voltammograms before and after the stress test is clearly shown in Supporting Information File 1, Figure S4. A decrease in the value of *E*_1/2_ is observed for all materials, but it is more pronounced for the samples G20 and JM20 and less pronounced for G30, G35, and G40 (Supporting Information File 1, Figure S4 and Table 3).

**Figure 8:**
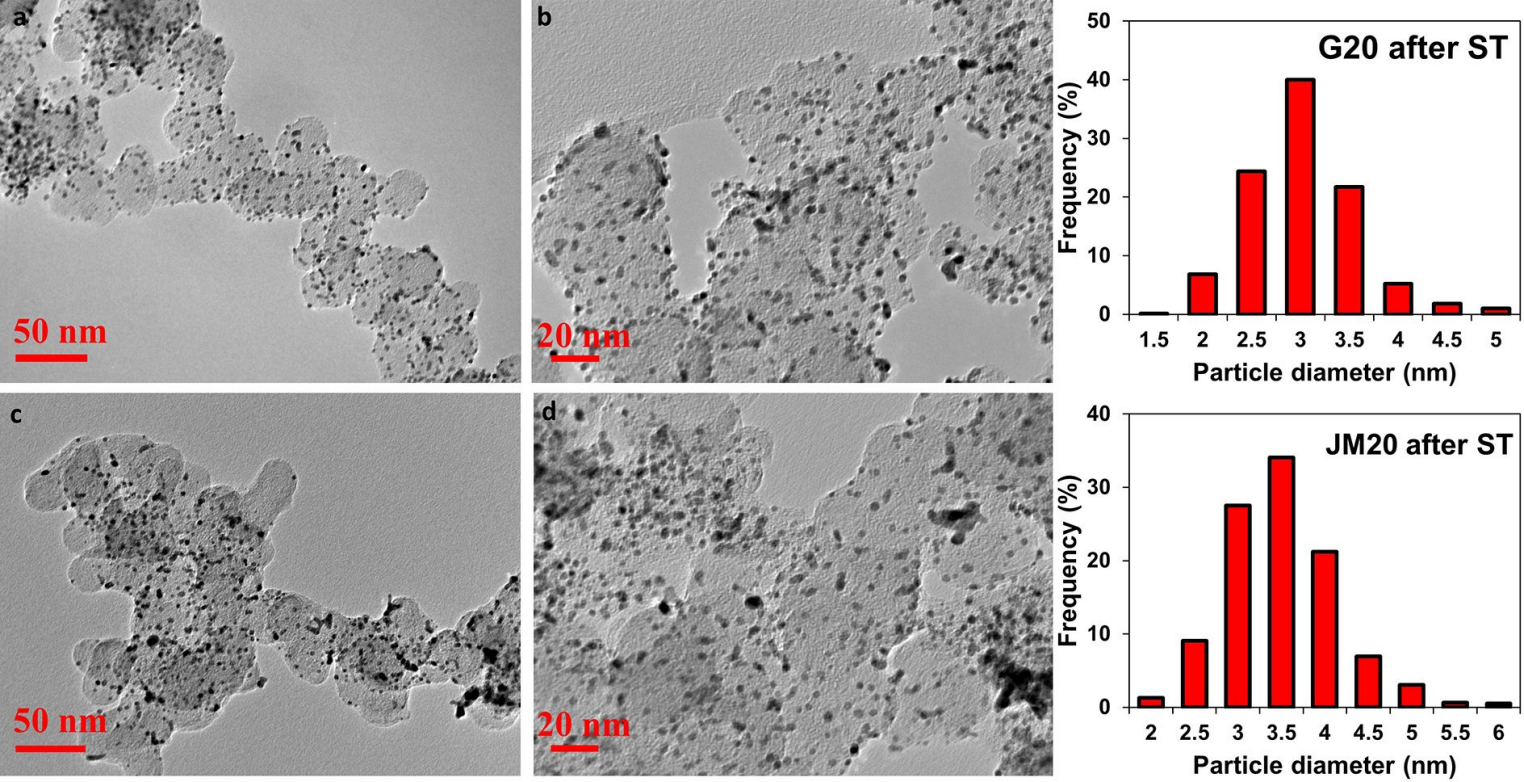
Micrographs of Pt/C samples of catalysts G20 (a,b) and JM20 (c,d) after stress test and histograms of nanoparticle size distribution of the corresponding materials.

The samples G20 and JM20 subjected to stress tests were examined by TEM. During the stress test the average nanoparticle size in the sample G20 increased from 2.0 to 3.0 nm, and in the sample JM20 it increased from 2.7 to 3.5 nm (Figure 8). The sample G20 also retained a narrower nanoparticle size distribution than that of JM20 (compare the histograms in Figure 8).

**Figure 9:**
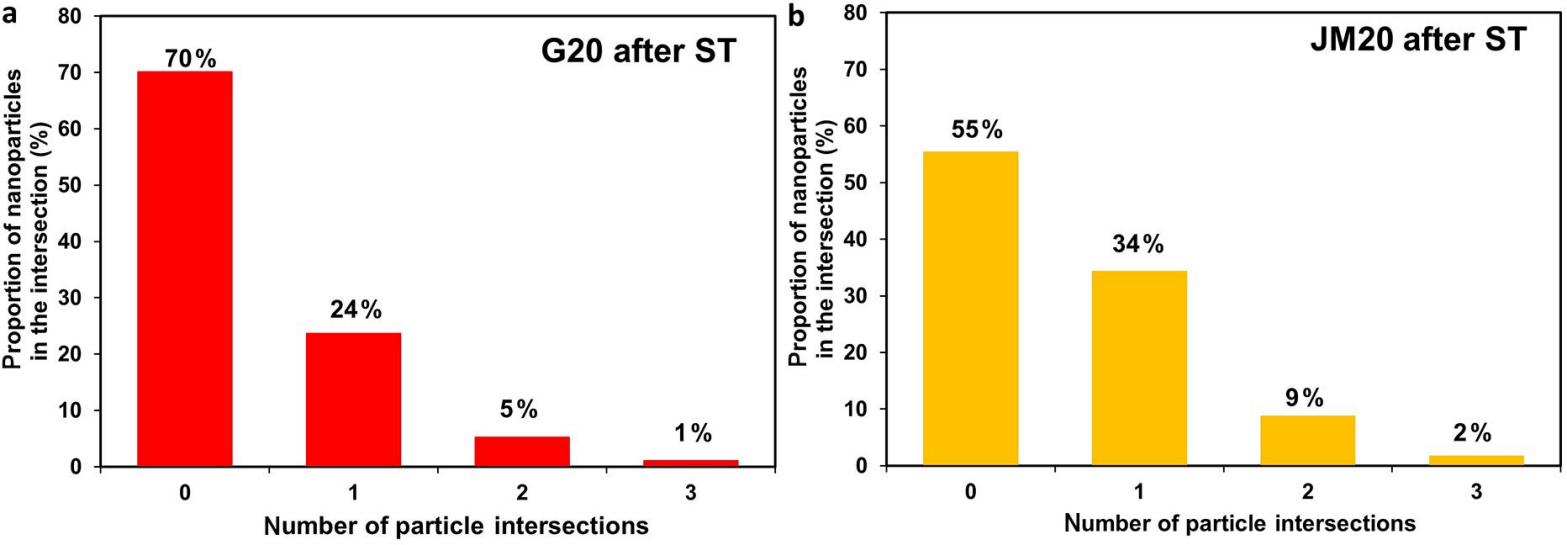
Histograms of nanoparticle distribution by the number of intersections with "neighbors" for the catalysts G20 (a) and JN20 (b) after the stress test.

The proportion of nanoparticles overlapping with one, two, or three neighbors was 45% in the sample JM20 and 30% in the sample G20 after the stress test (Figure 9).

Thus, even after the completion of the stress test, the sample G20 retained a more uniform morphology compared to the sample JM20. This result fully correlates with the results from the electrochemical behavior studies of the samples after the stress test.

Due to the changes in the structural characteristics of the catalysts, values of all three parameters in Equation 6 can change in a different way during the stress tests of each sample. This may be the reason for some change of the catalysts location in the row of increased mass activity, as compared to their location in the same row before the stress tests. Nevertheless, upon completion of the stress test, all the studied G series catalysts remained more active in the ORR compared to the commercial JM20 and JM40 catalysts.

The change in the ESA absolute values during the stress test, shown in Figure 7, is described by a linear dependence (see the correlation coefficients in Table 4). In the series of catalysts G20–G40 the rate of ESA reduction, calculated by the tangent of the slope formed by the straight lines, decreases with an increase in the Pt loading in the materials (Table 4). In this case, the values of the ESA rate decrease are very similar among catalysts with an identical Pt loading: G20 and JM20, G40 and GM40, respectively (Table 4). However, since the initial ESA for the catalysts G20 and G40 are significantly higher than that for JM20 and JM40, the synthesized catalysts G20 and G40 have a higher mass activity upon completion of the stress test than that of their commercial analogs JM20 and JM40.

**Table 4:** Coefficients of the slope angle formed by the straight line dependencies "ESA as a function of the cycle number" and the correlation parameters of the linear dependence equation.

sample	ω(Pt), wt %	−k × 10^6^, m^2^·g^−1^(Pt)	correlation coefficient, *R*^2^

G20	20.4	3691	0.882
G25	24.6	2982	0.959
G30	30.9	2945	0.987
G35	34.0	2855	0.938
G40	39.0	2545	0.888
JM20	20.0	3327	0.968
JM40	40.0	2655	0.984

## Conclusion

The Pt/C catalysts G20, G25, G30, G35, and G40 containing from 20 to 39 wt % of platinum with NPs of a small size (from 2 to 2.6 nm), which demonstrated a narrow and uniform size and spatial distribution on the surface and in the pores of the carbon support Vulcan XC-72, were obtained by liquid-phase synthesis.

Due to the smaller size and higher uniformity of the NP spatial distribution, the G series catalysts are characterized by higher ESA values (from 120 to 88 m^2^·g^−1^(Pt)) than the commercial Pt/C catalysts JM20 and JM40 (Johnson Matthey) (84 and 67 m^2^·g^−1^(Pt)), which contained 20 and 40 wt % of platinum, respectively. The ORR specific activity of all Pt/C catalysts turned out to be approximately the same. However, due to higher ESA values, the mass activity of G20 and G40 significantly exceeded the mass activity of the corresponding commercial samples JM20 and JM40.

The degradation of the catalysts was studied with the help of a stress test protocol, used in the "soft" mode, in which voltammetric potential sweep cycles were performed in the range 0.6–1.0 V. It was shown that the ESA decrease of catalysts during 5000 stress test cycles is fairly well described by a linear regression equation. The rate of ESA decrease reduces, as the Pt loading in the catalysts increases; however, it is almost identical for the synthesized and commercial catalysts with a similar Pt loading – G20 and JM20, G40 and JM40, respectively. After the stress tests, the mass activity of the catalysts decreases less than the ESA. Apparently, the degradation of the catalysts is not only due to a decrease in the ESA, but also due to a decrease in the fraction of the nanocrystal surface, which belongs to the regions with the highest catalytic activity. At the same time, the mass activity of the G series catalysts exceeds the mass activity of the commercial analogs both before and after the stress tests.

The results of this study show that Pt/C catalysts containing smaller nanoparticles can significantly exceed the catalysts based on larger nanoparticles in terms of ORR mass activity and they do not have an inferior stability. The reason for an unexpectedly high stability of catalysts containing small nanoparticles is most likely due to the uniformity of their structure, which manifests itself in the similarity of the nanoparticle sizes and uniformity of their distribution over the support surface. We have to admit that various methods for synthesizing Pt/C can also have an effect on the surface of the carbon support and the strength of the platinum NPs adhesion to its surface. Thus, the positive effects of the structural uniformity can compensate for the size effect that has a negative influence on the NP stability.

It is important to note that at a similar degradation rate (ESA decrease during the stress test) the Pt/C catalysts that we have synthesized demonstrated an ORR mass activity which exceeded the mass activity of the commercial Pt/C analogs by approx. 30–60%, both in the initial state and upon completion of the stress test.

## Supporting Information

This file shows how the average platinum crystallite size *D*_Av_ is calculated based on the X-ray diffraction pattern, how the electrochemically active surface area is obtained, how the catalytic activity is determined in the ORR, and the degradation degree of the electrocatalysts.

File 1 Experimental and theoretical methods used to obtain catalyst parameters.

## References

[1] Qin C, Wang J, Yang D, Li B, Zhang C (2016). Catalysts.

[2] O'Hayre R P (2009). Fuel cell fundamentals.

[3] Kongkanand A, Mathias M F (2016). J Phys Chem Lett.

[4] Katsounaros I, Cherevko S, Zeradjanin A R, Mayrhofer K J J (2014). Angew Chem, Int Ed.

[5] Chan S, Jankovic J, Susac D, Saha M S, Tam M, Yang H, Ko F (2018). J Mater Sci.

[6] Yaroslavtsev A B, Dobrovolsky Y A, Shaglaeva N S, Frolova L A, Gerasimova E V, Sanginov E A (2012). Russ Chem Rev.

[7] Litster S, McLean G (2004). J Power Sources.

[8] Jung N, Chung D Y, Ryu J, Yoo S J, Sung Y-E (2014). Nano Today.

[9] Borup R, Meyers J, Pivovar B, Kim Y S, Mukundan R, Garland N, Myers D, Wilson M, Garzon F, Wood D (2007). Chem Rev.

[10] Sui S, Wang X, Zhou X, Su Y, Riffat S, Liu C-j (2017). J Mater Chem A.

[11] Dai S, Zhang J, Fu Y, Li W (2018). J Mater Sci.

[12] Kinoshita K (1990). J Electrochem Soc.

[13] Gasteiger H A, Kocha S S, Sompalli B, Wagner F T (2005). Appl Catal, B.

[14] Meier J C, Galeano C, Katsounaros I, Witte J, Bongard H J, Topalov A A, Baldizzone C, Mezzavilla S, Schüth F, Mayrhofer K J J (2014). Beilstein J Nanotechnol.

[15] Garlyyev B, Kratzl K, Rück M, Michalička J, Fichtner J, Macak J M, Kratky T, Günther S, Cokoja M, Bandarenka A S (2019). Angew Chem, Int Ed.

[16] Alekseenko A A, Ashihina E A, Shpanko S P, Volochaev V A, Safronenko O I, Guterman V E (2018). Appl Catal, B.

[17] Rossi K, Asara G G, Baletto F (2020). ACS Catal.

[18] Hussain S, Erikson H, Kongi N, Sarapuu A, Solla-Gullón J, Maia G, Kannan A M, Alonso-Vante N, Tammeveski K (2020). Int J Hydrogen Energy.

[19] Leontyev I N, Belenov S V, Guterman V E, Haghi-Ashtiani P, Shaganov A P, Dkhil B (2011). J Phys Chem C.

[20] Holby E F, Sheng W, Shao-Horn Y, Morgan D (2009). Energy Environ Sci.

[21] Antolini E, Salgado J R C, Gonzalez E R (2006). J Power Sources.

[22] Cherevko S, Kulyk N, Mayrhofer K J J (2016). Nano Energy.

[23] Stamenkovic V R, Mun B S, Mayrhofer K J J, Ross P N, Markovic N M (2006). J Am Chem Soc.

[24] Capelo A, Esteves M A, de Sá A I, Silva R A, Cangueiro L, Almeida A, Vilar R, Rangel C M (2016). Int J Hydrogen Energy.

[25] Hasché F, Oezaslan M, Strasser P (2010). Phys Chem Chem Phys.

[26] Takei C, Kakinuma K, Kawashima K, Tashiro K, Watanabe M, Uchida M (2016). J Power Sources.

[27] Baschuk J J, Li X (2001). Int J Energy Res.

[28] Yan W-M, Chu H-S, Lu M-X, Weng F-B, Jung G-B, Lee C-Y (2009). J Power Sources.

[29] Park Y-C, Kakinuma K, Uchida M, Tryk D A, Kamino T, Uchida H, Watanabe M (2013). Electrochim Acta.

[30] Shao Y, Yin G, Gao Y (2007). J Power Sources.

[31] Shao-Horn Y, Sheng W C, Chen S, Ferreira P J, Holby E F, Morgan D (2007). Top Catal.

[32] Xie J, Wood D L, More K L, Atanassov P, Borup R L (2005). J Electrochem Soc.

[33] Guterman V E, Belenov S V, Alekseenko A A, Tabachkova N Y, Volochaev V A (2017). Russ J Electrochem.

[34] Moguchikh E A, Alekseenko A A, Guterman V E, Novikovsky N M, Tabachkova N Y, Menshchikov V S (2018). Russ J Electrochem.

[35] Zhang Y, Chen S, Wang Y, Ding W, Wu R, Li L, Qi X, Wei Z (2015). J Power Sources.

[36] Guterman V E, Belenov S V, Alekseenko A A, Lin R, Tabachkova N Y, Safronenko O I (2018). Electrocatalysis.

[37] Watanabe M, Yano H, Uchida H, Tryk D A (2018). J Electroanal Chem.

[38] Dendooven J, Ramachandran R K, Solano E, Kurttepeli M, Geerts L, Heremans G, Rongé J, Minjauw M M, Dobbelaere T, Devloo-Casier K (2017). Nat Commun.

[39] Yano H, Watanabe M, Iiyama A, Uchida H (2016). Nano Energy.

[40] Alinejad S, Quinson J, Schröder J, Kirkensgaard J J K, Arenz M (2020). ACS Catal.

[41] Favilla P C, Acosta J J, Schvezov C E, Sercovich D J, Collet-Lacoste J R (2013). Chem Eng Sci.

[42] Gusev A I (2009). Nanomaterials, nanostructures, nanotechnologies.

