# Supplementary material for: Stability and activity of platinum nanoparticles in the oxygen electroreduction reaction: is size or uniformity of primary importance?
Source: Beilstein J Nanotechnol. 2021 Jun 29;12:593–606. doi: 10.3762/bjnano.12.49 (PMC8261527; doi:10.3762/bjnano.12.49)
Supplement: File 1 — Experimental and theoretical methods used to obtain catalyst parameters. [file Beilstein_J_Nanotechnol-12-593-s001.pdf]

BEILSTEIN JOURNAL OF NANOTECHNOLOGY

# Supporting Information

for

# Stability and activity of platinum nanoparticles in the oxygen electroreduction reaction: is size or uniformity of primary importance?

Kirill O. Paperzh, Anastasia A. Alekseenko, Vadim A. Volochaev, Ilya V. Pankov, Olga A. Safronenko and Vladimir E. Guterman

*Beilstein J. Nanotechnol.* **2021,** *12,* 593–606. doi:10.3762/bjnano.12.49

# Experimental and theoretical methods used to obtain catalyst parameters

License and Terms: This is a supporting information file under the terms of the Creative Commons Attribution License (https://creativecommons.org/licenses/by/4.0). Please note that the reuse, redistribution and reproduction in particular requires that the author(s) and source are credited and that individual graphics may be subject to special legal provisions.
The license is subject to the *Beilstein Journal of Nanotechnology* terms and conditions: (https://www.beilstein-journals.org/bjnano/terms)

# X-ray study

The average platinum crystallite size $D_{Av}$ was calculated using the Scherrer equation [1].

$$D_{Av} = K\lambda / (\mathrm{FWHM} \cos\theta), \quad \text{(S1)}$$

where λ is the wavelength of the monochromatic radiation (in Å), FWHM is the reflex half width at half maximum (in radians), K = 0.89 is the Scherrer's constant), $D_{Av}$ is the average thickness of the "stack" of reflecting planes in the coherent scattering region (in Å), and θ is the angle of incidence of the X-ray beam (in radians).

An example of processing the X-ray diffraction pattern of a Pt/C catalyst using the SciDavis program, which makes it possible to refine the calculation of the $D_{Av}$, is shown in Figure S1.

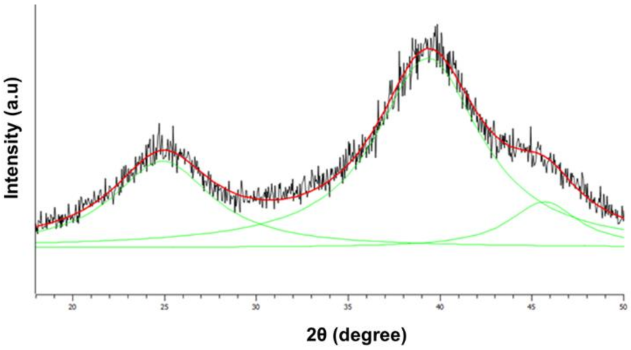


**Figure S1:** An example of X-ray diffractogram fitting using the SciDavis software.

# Determination of the electrochemically active surface area of the catalysts by cyclic voltammetry

The electrochemically active surface area (ESA) was calculated from the charge amount consumed for desorption $Q_d$ and adsorption $Q_{ad}$ of hydrogen using the formula:

$$\mathrm{ESA} = ((Q_d + Q_{ad}) \cdot 0.5) / (210 \cdot m(\mathrm{Pt}) \cdot 1000), \quad (2)$$

where ESA is the area of electrochemically active platinum surface ($m^2 \cdot g^{-1}$(Pt)), $Q_d$ and $Q_{ad}$ (i.e., the charge amount consumed for electrochemical desorption and adsorption of atomic hydrogen, respectively, (μC) (Figure S2)), 210 is the charge required for the formation/oxidation of an atomic hydrogen monolayer per 1 $cm^2$ of platinum surface (μC $cm^{-2}$), m(Pt) is the platinum weight on the electrode (g).

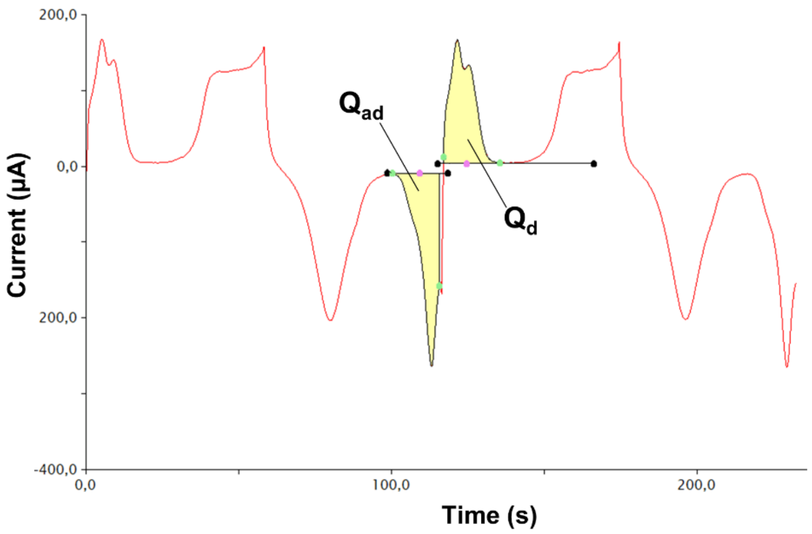


**Figure S2:** Current as a function of time during CV recording. The areas on the graph are highlighted for the calculation of the charge amount: $Q_{ad}$ - the amount of electricity spent on atomic hydrogen adsorption; $Q_d$ is the amount of electricity consumed for atomic hydrogen desorption.

The charge amount was determined from the cyclic voltammogram (CV) as the area under the line of the current–time dependence in the potential range of 0.04–0.40 V minus the contribution of the double-layer region.

# Determination of the catalytic activity in the ORR

To take into account the contribution of the ohmic potential drop and processes not associated with ORR, the voltammograms obtained when the potential was swept towards more positive values (Figure S3) were normalized according to generally accepted techniques, as presented below in more detail. For this, the potential of the electrode under study was refined using the formula:

$$E = E_{\text{set}} - I \cdot R, \quad (3)$$

where $E_{\text{set}}$ is the set value of the potential, $I{\cdot}R$ is the ohmic potential drop equals to the product of the current ($I$) and the resistance ($R$) of the solution layer between the reference and work electrodes, which was 23 Ω [2].

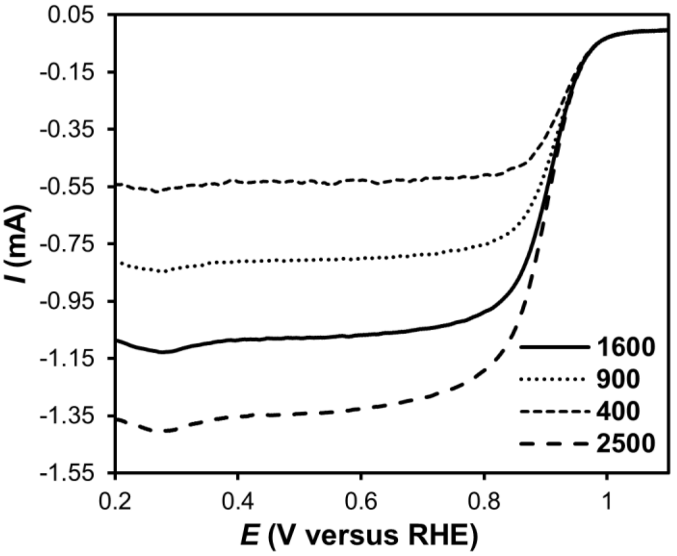


**Figure S3:** Potentiodynamic polarization curves of ORR at different speed values of the disk rotation. 0.1 M $HClO_4$. $O_2$ atmosphere. Potential speed rate is 20 mV·s$^{-1}$.

The contribution of the processes occurring at the electrode in the deoxygenated solution (Ar atmosphere) was taken into account by subtracting from the voltammogram a similar curve recorded at the same electrode during measurements in an Ar: $I(O_2) - I(\text{Ar})$ atmosphere. The ORR activity of the catalysts (kinetic current) was determined from normalized

voltammograms taking into account the contribution of mass transfer under the conditions of RDE [3].

The kinetic current was calculated using the Koutetskiy–Levich equation:

$$1/j = 1/j_k + 1/j_d, \tag{4}$$

where $j$ is the experimentally measured current, $j_d$ is the diffusion current, and $j_k$ is the kinetic current. The kinetic currents were calculated for a potential of 0.90 V (RHE). Dividing the kinetic current by the mass of platinum loaded onto the electrode and the ESA, we obtained such activity parameters as mass ($I_{mass}$) and specific ($I_{sp}$) currents, respectively. The number of electrons involved in ORR was determined by the angle of the line in coordinates $1/j - 1/\omega^{0.5}$.

## Evaluation of the degradation degree of the electrocatalysts in the process of multiple cycling

The stability was assessed by the ratio of platinum ESA values after ($ESA_j$) and before ($ESA_0$) the stress test using the formula:

$$\text{Stability} = \left(\text{ESA}_j / \text{ESA}_0\right) \cdot 100\%, \tag{5}$$

where j is the number of potential sweep cycles carried out before a particular measurement of ESA.

The degree of degradation (DD) was calculated using the formula:

$$\text{DD} = \left(1 - \text{ESA}_j / \text{ESA}_0\right) \cdot 100\%. \tag{6}$$

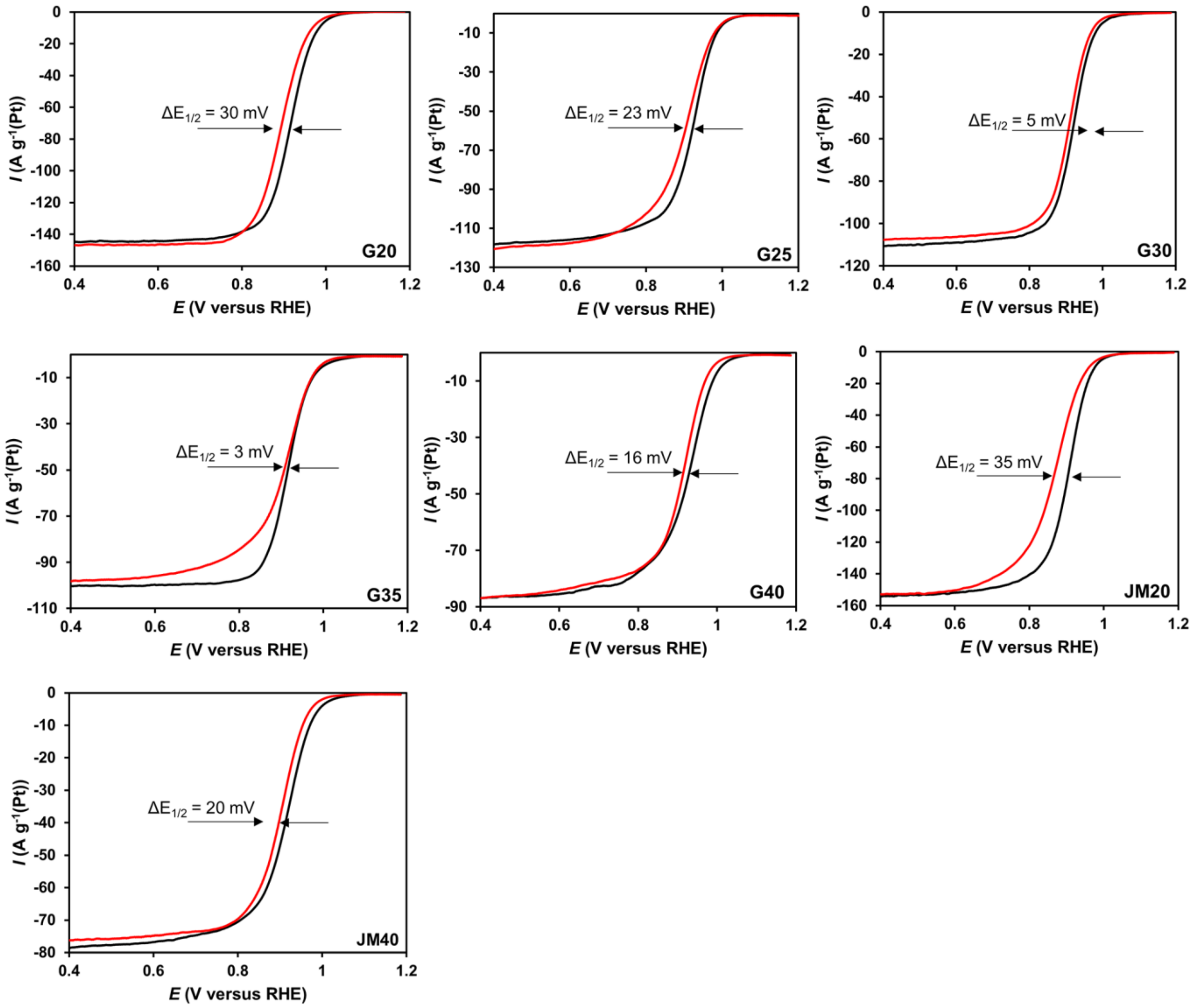


**Figure S4:** Potentiodynamic polarization curves of ORR before the start (black) and after completion (red) of stress testing. The rotation speed of the RDE is 1600 rpm. 0.1 M $HClO_4$. $O_2$ atmosphere. The potential sweep rate is 20 mV·s$^{-1}$.

# References:

1. Langford JI (1978) Scherrer after sixty years: A survey and some new results in the determination of crystallite size. J Appl Crystallogr 11:102–113. doi: 10.1107/S0021889878012844

2. Shinozaki K, Zack JW, Pylypenko S, Pivovar BS, Kocha SS (2015) Oxygen reduction reaction measurements on platinum electrocatalysts utilizing rotating disk electrode

technique: II. Influence of ink formulation, catalyst layer uniformity and thickness. J Electrochem Soc 162:F1384–F1396. doi: 10.1149/2.0551512jes

3. Hyun K, Lee JH, Yoon CW, Kwon Y (2013) The effect of platinum based bimetallic electrocatalysts on oxygen reduction reaction of proton exchange membrane fuel cells. Int J Electrochem Sci 8:11752–11767
